# Marine biotechnology: diving deeper for drugs

**DOI:** 10.1111/1751-7915.12410

**Published:** 2016-09-06

**Authors:** Detmer Sipkema

**Affiliations:** ^1^ Laboratory of MicrobiologyWageningen UniversityStippeneng 46708 WEWageningenThe Netherlands

## Abstract

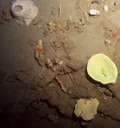

‘That which does not kill us, makes us stronger’ was Friedrich Nietzsche's quote in his *Twilight of the Idols*. Yet, a conservative estimation is that antibiotic resistance now annually attributes to 700 000 deaths globally, with a potential leap to 10 million in 2050 (O'Neill, 2014). In addition, a global surveillance report issued by the WHO indicated that infectious diseases due to antibiotic resistance could lead to a world‐wide economic loss of 100 trillion US$ in 2050 (WHO, 2014). It is therefore likely that Nietzsche's immature advice will not make us stronger, but rather put us back to the age of the Austro‐Hungarian phycisian Ignaz Semmelweis, who advocated hand washing to prevent infections (Semmelweis, 1861). Semmelweis’ methods to prevent bacterial infections were hugely reinforced by Flemming's rather serendipitous discovery of penicillin (Flemming, 1929). This was the forebode of a golden age of discovery of natural antibiotics and semi‐synthetic derivatives in the 1950s and marked a new era of health care in which bacterial pathogens could be treated effectively (Davies and Davies, 2010). However, since that period, there has been a slump in the development of antibiotics and the discovery of teixobactin from a soil bacterium in 2015 has been called the first new antibiotic scaffold in 30 years (Ling *et al*., 2015). This decline in antibiotics discovery has coincided with increased antibiotic resistance of pathogens and holds an emerging global threat with the return of microbial foes that were once slain. The consequence is that that minor cuts and scrapes, as well as medical interventions from common caesarean sections to complex organ transplantations render patients very vulnerable to infections by antibiotic‐resistant bacteria.

Although development of *new* antibiotic scaffolds is not the only intervention needed, it is crucial to find novel ‘last resort’ antibiotics to treat patients infected with multidrug‐resistant bacteria. The quest for new molecules with pharmaceutical properties has seen a shift away from large synthetic combinatorial libraries and a return to nature during the last decade. Conceptual and technological developments in ‘omics’ have given a new incentive to scrutinize nature because genes and proteins of the uncultivated majority of microorganisms are now available. ‘Omics’ analyses of microbes, plants and marine invertebrates, coupled with synthetic biology techniques, promise to open the world's treasure trove of natural products at a rate that would outpace antibiotic discovery at its peak in the 1950s (Fortman and Mukhopadhyay, 2016). A promising strategy to re‐tap the reservoir of natural antibiotics is to shift focus from the terrestrial to the marine environment, which is despite a growing number of studies still largely *mare incognita*. SCUBA diving has greatly increased access to the marine environment, but we have merely scratched the shallow parts. It has been said that we know more about the surface of the Moon than about the bottom of our oceans (Snelgrove, 2011), but in fact we do not need to go very deep for us to be rather ignorant about the biology and the molecules that can be found. SCUBA divers can reside only briefly at a depth of 40 m and the large majority of the divers has a license for only 20 m below sea level. It should therefore be no surprise that we have mostly been studying the ‘tip of the iceberg’. A recent landmark (seamark) study showed that richness of functional genes in seawater samples increased with depth when samples collected at 5, 71 and 600 m were compared (Sunagawa *et al*., 2015). In addition, when the genes found at these different depths were compared to genetic data present in public databases, a stunning number of 90% of the genes detected at 600 m did not have a match in public databases (Sunagawa *et al*., 2015).

An interesting divide between the terrestrial and the marine environment is that, while the Earth's surface on land is mostly covered with plants, the benthic organisms in the oceans are mainly sessile animals that use chemical warfare to fight for space and protect themselves. Marine invertebrates have been identified as one of the most promising natural sources for future antibiotics (Fortman and Mukhopadhyay, 2016) and particularly sponges have repeatedly been called the most prolific source of natural products with more than 7000 bioactive compounds identified (Blunt *et al*., 2016; Indraningrat *et al*., 2016). Based on my own subjective observations during submarine expeditions at Curaçao and Dominica (Fig. 1) it was striking to see that the diverse benthic communities of marine invertebrates in the mesophotic region at 100–150 m were apparently nearly all different species than the ones seen at SCUBA depth (0–40 m). Many of the sponges found in the mesophotic zone turned out to be indeed species yet undescribed (Van Soest *et al*., 2014) and it appears that deep‐sea sponges harbour a microbiome and secondary metabolite biosynthetic gene clusters that differ from their shallow‐water counterparts (Kennedy *et al*., 2014; Borchert *et al*., 2016). It would therefore be surprising if we would *not* find entirely new mechanisms and molecules related to antibiotics in the deep biological dark matter. The predicted and partly unveiled novelty of the large majority of the genes and species in the deep sea enhances the chances to not only discover variations on a theme, but truly new scaffolds for the development of new ‘last resort’ antibiotics. Nietzsche would only agree on the high expectations of discovering new ‘gold’ in the sea: ‘From the Sun I learned this: when he goes down, over rich; he pours gold into the sea out of inexhaustible riches, so that even the poorest fisherman still rows with golden oars’ (Nietzsche, 1883).

**Figure 1:**
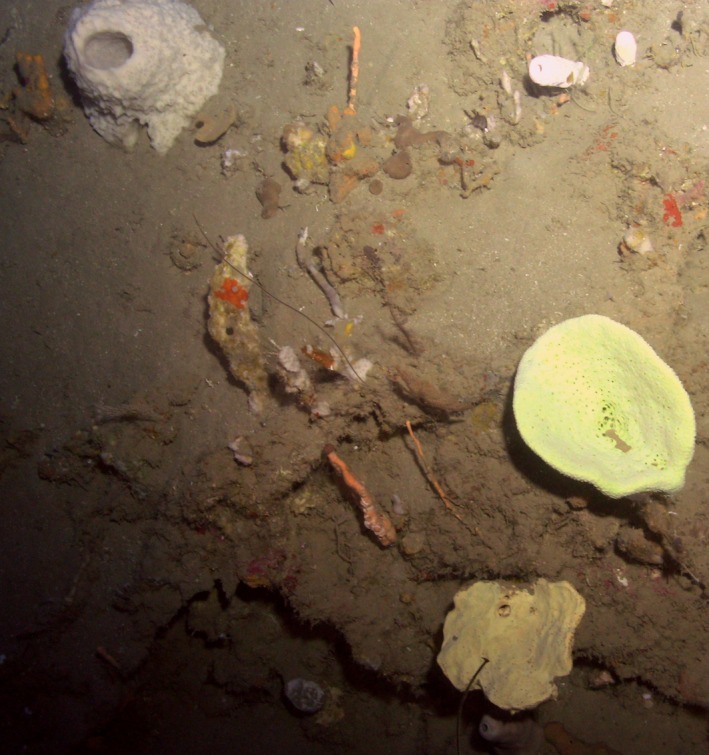
A sponge ground near Dominica at a depth of 100 m. Picture made by Substation Curaçao.

## Conflict of interest

None.
